# Aging, Culture, and Memory for Socially Meaningful Item-Context Associations: An East-West Cross-Cultural Comparison Study

**DOI:** 10.1371/journal.pone.0060703

**Published:** 2013-04-11

**Authors:** Lixia Yang, Juan Li, Julia Spaniol, Lynn Hasher, Andrea J. Wilkinson, Jing Yu, Yanan Niu

**Affiliations:** 1 Department of Psychology, Ryerson University, Toronto, Ontario, Canada; 2 Center on Aging Psychology, Key Laboratory of Mental Health, Institute of Psychology, Chinese Academy of Sciences, Beijing, China; 3 Department of Psychology, University of Toronto, Toronto, Ontario, Canada; 4 The Rotman Research Institute, Toronto, Ontario, Canada; University of Nottingham Malaysia Campus, Malaysia

## Abstract

Research suggests that people in Eastern interdependent cultures process information more holistically and attend more to contextual information than do people in Western independent cultures. The current study examined the effects of culture and age on memory for socially meaningful item-context associations in 71 Canadians of Western European descent (35 young and 36 older) and 72 native Chinese citizens (36 young and 36 older). All participants completed two blocks of context memory tasks. During encoding, participants rated pictures of familiar objects. In one block, objects were rated either for their meaningfulness in the independent living context or their typicality in daily life. In the other block, objects were rated for their meaningfulness in the context of fostering relationships with others or for their typicality in daily life. The encoding in each block was followed by a recognition test in which participants identified pictures and their associated contexts. The results showed that Chinese outperformed Canadians in context memory, though both culture groups showed similar age-related deficits in item and context memory. The results suggest that Chinese are at an advantage in memory for socially meaningful item-context associations, an advantage that continues from young adulthood into old age.

## Introduction

Research suggests that people from different cultures perceive the world in different ways and that culture at least partially shapes information processing styles. Several researchers have proposed that accumulated cultural experience (e.g., socialization, language acquisition, and parent-child interactions) guides our attention to select some aspects of information (e.g., focal objects or contexts) over others for processing and subsequent memory [1], [2]. Specifically, individuals from Western cultures (e.g., North America) tend to view the world in an analytic way and attend to object-based information, whereas those from East Asian cultures (e.g., Japan, China, and Korea) view the world in a holistic way and attend to contextual details [3]–[8].

These findings suggest that East Asians may outperform Westerners with respect to memory for item-context associations. Previous cross-cultural studies have provided mixed evidence for cultural differences in item-context binding. A number of studies have shown that East Asians prioritize contextual information, whereas Westerners prioritize object-based information in processing complex scenes [9]–[11]. For example, in a study testing memory for complex scenes, Masuda and Nisbett [11] found that Japanese participants remembered more background information than did Americans, though the two groups showed equivalent memory for central objects. Furthermore, object recognition was more impaired in Japanese than in Americans by changing or removing associated background. These findings suggest that East Asians may bind items to associated contexts more readily than Westerners.

In contrast, other studies failed to detect cultural differences in item-context binding at either behavioral or neural levels. For example, Goh et al. [12] tested young and older Singaporeans and Americans using a functional magnetic resonance adaptation (i.e., fMR-adaptation) paradigm in which participants passively viewed four complex scene pictures, with the focal object and/or background systematically repeated or varied on each trial. Neural adaptation involved in the processing of objects, backgrounds, and object-background associations was indexed by the attenuation of the blood oxygen level dependent (BOLD) signal in response to the corresponding repetitions. The findings showed few culture differences in neural regions and adaptation in processing background and object-background associations [12]. Similarly, in a study comparing young and older Chinese and American adults, Chua and colleagues [13] found culturally-equivalent source memory for four speakers who introduced specific statements during the study phase [13].

We note that in the studies by Goh et al. [12] and Chua et al. [13], the item-source pairings were arbitrarily assigned and the binding process merely relied on passive viewing or listening at a more feature-driven perceptual level. Memory for these types of associations may be highly sensitive to changes in biological or neurocognitive resources that occur with aging universally across cultures [14]. Using the same fMR-adaptation paradigm as in Goh et al. [12], a recent study showed that Chinese were more sensitive to contextual incongruity at a semantic level, as indexed by their larger neural adaptation to incongruent (e.g., a cow in a kitchen scene) than to congruent scenes (e.g., a cow in a farm scene), in comparison with Americans [15]. However, few studies have examined cultural effects on memory for actively processed socially meaningful item-context associations, despite promising evidence for cultural differences in processing meaningful background scenes [11], [12]. Furthermore, most North American studies suggest an age-related deficit in explicit memory for item-context associations [16]. However, little is known whether this decline applies equally to East Asians.

In the current study, we sought to investigate the effects of culture and age on memory for socially meaningful item-context associations formed through actively rating items for their meaningfulness or typicality in various social contexts. To engage socially meaningful associative processing, we used an intentional context memory paradigm. Participants studied pictures of everyday objects and rated their meaningfulness or typicality in different social contexts, and were then tested on their memory for pictures and associated contexts. The social context was manipulated to favor either Eastern or Western culture based on the widely reported assumption that Chinese place great value on interpersonal relationships whereas Westerners tend to value individual autonomy [17]–[19]. In the “RELATIONAL” (R) condition, participants rated how meaningful/useful each object was for “you to get along with others and to be liked and accepted by others” in a new city. This social context emphasizes interdependent collectivism (e.g., attending to others and harmonious interdependency) which is typical in East Asian cultures [19]. In the “INDEPENDENT” (I) condition, participants rated how meaningful/useful each object was for “you to live independently on your own” in a new city. This condition emphasizes individual autonomy and independence believed to be highly valued by Westerners [19]. During encoding, participants rated pictures of familiar objects in two separate blocks delivered in a counterbalanced order, each followed by a recognition test. In one block (i.e., the I/D block), objects were rated either for their meaningfulness in the independent living context (I) or their typicality in daily life (DAILY LIFE context, D). In the other block, objects were rated for their meaningfulness in the context of fostering relationships with others (R) or for their typicality in daily life (i.e., the R/D block). This way, the R and I contexts were each paired with a culturally neutral but socially meaningful context, D. The R and I conditions were not presented in the same block in order to minimize the possibility that participants would employ a recall-to-reject heuristic (e.g., I know that this item was not encoded in context A, so it must have been encoded in context B) [20], and to increase the likelihood of recollection-based context retrieval.

If cultural differences in socially-meaningful item-context associations do exist, they could be either context-independent or context-specific. In the context-independent case, Chinese should outperform Canadians in context memory across different types of contexts. Specifically in this study, we would expect an overall memory advantage for Chinese relative to Canadians for both I and R contexts. In the context-specific case, Chinese would outperform Canadians only in memory for their culturally appropriate contexts (e.g., R context in this study). In either case, the context memory advantage of Chinese is presumably driven by their culture-based processing preference for contextual information [11]. This experience-based cultural effect may be preserved or even magnified in older adults, relative to young adults, due to life-long experience with culturally-preferred cognitive pragmatics [14], [21], [22].

The context memory paradigm also allowed us to examine potential cultural effects on memory for items. Item memory, independent of item-context associations, is believed to tax basic, culturally-invariant cognitive functions [7], [14]. As such, we expected that item memory performance would not differ between Chinese and Canadian participants, and that both cultures would show similar age-related deficits.

In summary, the current study examined cultural and age differences in memory for socially meaningful item-context associations. It aimed to address three specific questions: 1) Do Chinese and Canadians differ in their memory for socially meaningful item-context associations? 2) Is the cultural effect context-independent (i.e., across different social contexts) or context-specific (i.e., specific to culturally preferred social context)? 3) Does the cultural effect differ between young and older adults? Based on previous findings, we hypothesized that 1) Chinese would outperform Canadians in memory for socially meaningful item-context associations; 2) The cultural effect would be either context-independent (i.e., for both I and R contexts) or context-specific (i.e., only for R context); and 3) the cultural effect would be preserved or even magnified with aging.

## Methods

### Participants

The sample consisted of 71 Canadians, including 35 young (ages 18–28, with 27 females and 8 males) and 36 older adults (ages 60–80, with 14 females and 22 males), and 72 Chinese, including 36 young (ages 18–25, with 19 females and 17 males) and 36 older adults (ages 60–75, with 20 females and 16 males). The detailed sample characteristics and related statistics are displayed in Table 1. The overall *χ* ^2^ test showed that the two cultures did not differ in gender distribution, *χ^2^* = 0.19, *p* = .67. All Canadians were of Western European descent. Young Canadians were recruited from an undergraduate participant pool in Toronto whereas young Chinese were undergraduate students recruited through campus posters from universities in Beijing. Older Canadians and Chinese were recruited through an internal participant pool and/or through posters from local communities in Toronto and Beijing.

**Table 1:** The Sample Characteristics.

	Canadian		Chinese		*F* values		
	Young	Older	Young	Older	Age	Culture	Interaction
	(n = 35)	(n = 36)	(n = 36)	(n = 36)			
Age	20.77 (2.95)	68.86 (6.03)	20.69 (1.56)	67.72 (4.47)	4775.32**		
Education^a^	14.01 (2.23)	16.28 (3.33)	14.06 (1.17)	14.47 (2.66)	10.48**	4.54*	4.98*
Health^b^	7.81 (1.11)	8.41 (1.35)	7.82 (1.22)	7.58 (1.27)		3.93*	4.03*
Independent^c^	4.96 (0.66)	5.34 (0.72)	4.49 (0.57)	4.99 (0.75)	15.08**	13.15**	
Interdependent^c^	4.72 (0.54)	4.53 (0.68)	5.01 (0.59)	5.37 (0.58)		31.30**	7.66**
VSWM^c^	59.05 (16.34)	27.31 (14.99)	66.67 (18.69)	32.87 (14.77)	144.96**	5.86*	
CES-D	14.31 (9.76)	8.28 (8.02)	14.36 (7.15)	5.17 (5.43)	34.68**		
MMSE	N/A	28.86 (1.17)	N/A	28.86 (1.10)			

*Note.* Only significant *F* values are reported.

* *p*<.05.

** *p*<.01.

Each cell provides mean score, with standard deviation in parenthesis. VSWM = visual-spatial working memory; CES-D = the Center for Epidemiological Studies Depression Scale; MMSE = the Mini-Mental State Examination.

a Education was measured in years of formal education;

b Health was measured by self-report ratings on a 1–10 Likert-Type scale.

c Independent and interdependent self-construal scores on the SCS.

Young Canadians received course credits and all the other groups received monetary compensation for their participation. One young Canadian was excluded from the final sample due to a false alarm rate of 100%, reflecting a possible misunderstanding or confusion of response keys during recognition. Three older Chinese and eight Canadian participants (five young and three older) were replaced due to health-related or administration-related problems, or failure to properly follow the instructions.

Verbal skills were measured to ensure language proficiency. All Canadian participants scored above 20 on the Shipley vocabulary test [23], with a higher average score for older (*M* = 36.14, *SD* = 3.45) than for young adults (*M* = 28.77, *SD* = 4.59), *t*(69) = 7.66, *p*<.001. All Chinese participants scored above 41 on the vocabulary subtest of the Wechsler’s Adult Intelligence Scale [24], and the two age groups did not differ (young: *M* = 61.94, *SD* = 7.74; older: *M* = 62.44, *SD* = 7.45), *t*(70) = 0.28; *p = *.78. All older adults scored above 26 on the Mini-Mental State Examination (MMSE) [25], a screening test for potential cognitive impairment. The two cultures did not differ on MMSE scores, *t*(70) = 0.00; *p = *1.00. This suggested that older adults across the two cultures were matched on basic cognitive functioning.

To assess potential covariates of memory performance and maximally match the participants across age and culture groups, we administered a demographic questionnaire, the Center for Epidemiological Studies Depression Scale (CES-D) [26], and the Self-Construal Scale (SCS) [27]. A 2 (age)×2 (culture) ANOVA was conducted on each of the resulted variables (see Table 1 for *F* values). In both cultures, older adults scored lower on the CES-D (i.e., less depressed), and higher on the SCS independent self-construal than did young adults. For older but not young adults, Canadians had more years of education and rated themselves as healthier than Chinese. Finally, Canadians scored higher on the SCS independent self-construal, but lower on the SCS interdependent self-construal than did Chinese, suggesting that our samples well represent their own cultural norms [28]. The cultural effect on the interdependent self-construal tended to be larger for older than for young adults. Despite cultural and age differences in all of these variables, the main age and/or cultural effects on item and context memory scores reported below in the results section remained significant in covariance analyses after controlling for all the variables that showed age or cultural differences: education, health-rating, the SCS scores and the CES-D scores. These findings suggest that the age and cultural differences in these variables do not account for the main age or cultural effect on memory.

### Ethics Statement

The present study was approved by the Research Ethics Board of Ryerson University in Canada as well as the Research Ethics Committee of the Institute of Psychology, Chinese Academy of Sciences, in China. Written informed consent was obtained from each participant.

### Stimuli

A total of 180 line-drawing pictures of common objects were chosen from a database [29] that has been normed with American and Chinese young and older adults [30]. All the objects in the chosen pictures are easily recognizable objects that are relatively familiar to people from both cultures (familiarity ratings ranged from 2.4–5 based on a 5-point scale, with larger numbers denoting higher familiarity). The pictures were divided into six sets of 30 each. The sets did not differ in familiarity ratings across the four Age × Culture groups, *F* (174) = 0.08; *p* = .995. The sets were counterbalanced across participants, such that each set was assigned equally often to each of the two context memory blocks. Within each block, an encoding task was followed by a recognition task. Each set was encoded equally often in the two contexts (I/R or D) or served as the new set at recognition.

### Procedure

**Figure 1:**
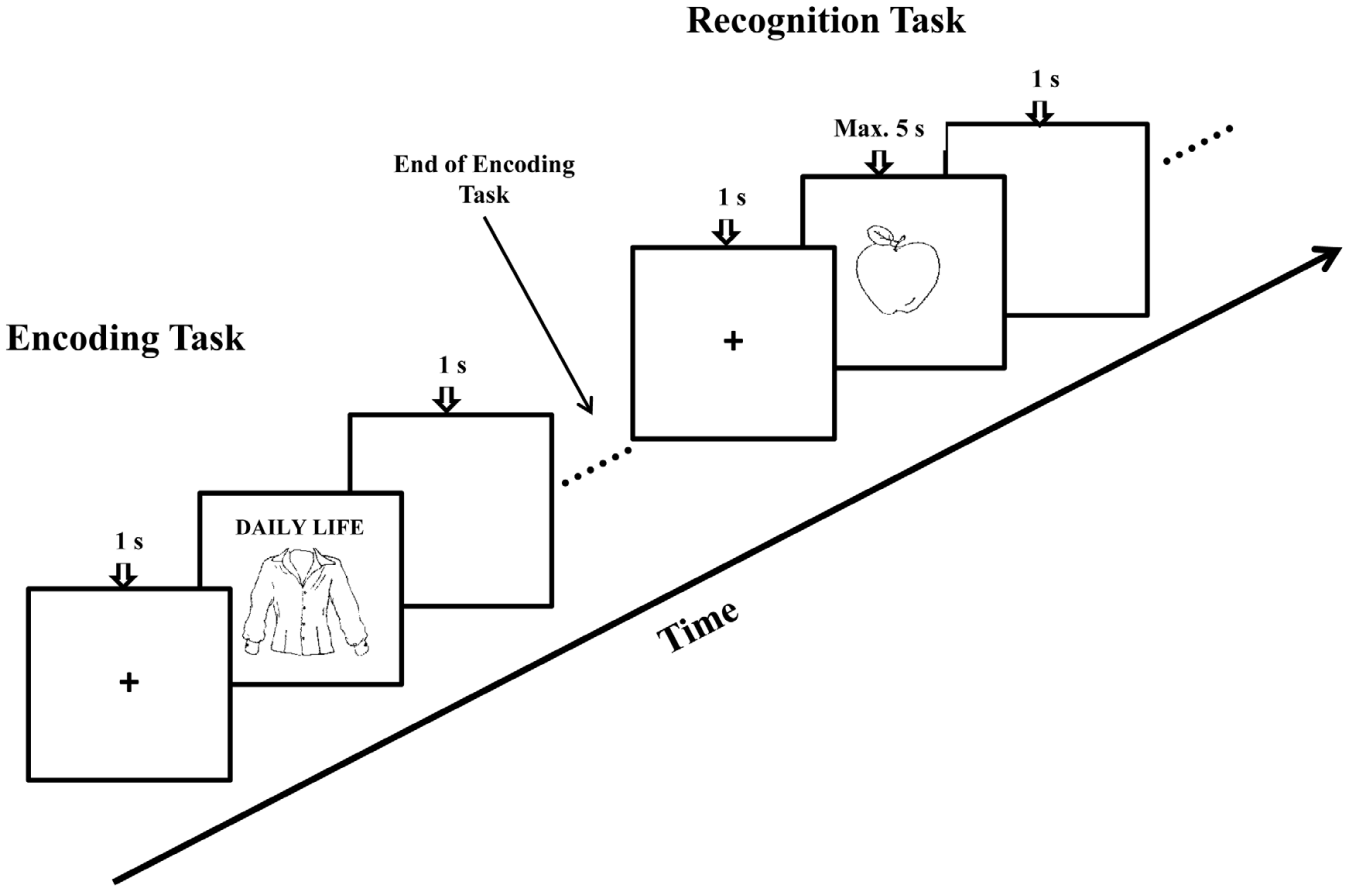
A sample trial procedure at encoding and retrieval (i.e., recognition task) within each context memory block. The cue word presented above the picture during encoding was either “DAILY LIFE” or “INDEPENDENT” (half-half) in the I/D block; whereas the cue word was either “DAILY LIFE” or “RELATIONAL” (half-half) in the R/D block. The encoding task was self-paced in that each stimulus stayed until a response was given.

After signing the consent form, participants completed two blocks of the context memory tasks (i.e., the I/D and the R/D blocks), in a counterbalanced order. Within each block, the encoding task was followed by a recognition task. The trial procedure for both encoding and retrieval is illustrated in Figure 1. A computerized visual-spatial working memory (VSWM) task (i.e., a mouse version modified from [31]) was used as a filler task between the two memory blocks to reduce potential interference from the first block and thus ensure an effective switch to a new set of encoding instructions.

In each context memory block, participants viewed a series of 60 centrally presented object pictures, each accompanied by a cue word presented above. Half of the pictures were cued with “INDEPENDENT” (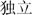 in Chinese) or “RELATIONAL” (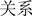 in Chinese), and the other half were cued with “DAILY LIFE” (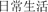 in Chinese). For the I or R context, participants were instructed to imagine that they were moving to a new city, and to rate how meaningful each object would be “for you to live independently on your own” (i.e., I) or “for you to get along with others and to be liked and accepted by others” (i.e., R) in the new city. Ratings were based on a scale of 1 (“least meaningful”) to 5 (“most meaningful”). For the D context, participants were instructed to rate how typical the object was in daily life based on a similar scale of 1 (“least typical”) to 5 (“most typical”). The pictures were presented in a pseudo-randomized order so that no more than three pictures with the same context cue appeared consecutively. Responses were made by pressing the corresponding number keys. Each trial started with a centered fixation cross (1 s), followed by a stimulus (i.e., a picture with a cue word presented above). The stimulus stayed on the screen until a response was given (i.e., self-paced responses). Following each response, a blank screen was presented for 1 s as an inter-stimulus interval (ISI) before proceeding to the next trial. Prior to the experimental trials, participants completed four practice trials (two in the I or R and two in the D contexts).

The encoding task in each block was followed by a recognition task in which participants viewed 90 pictures (60 studied and 30 non-studied new) and responded to each by pressing one of three different keys to indicate the picture was old studied in I or R context, old studied in D context, or a new picture. Each trial started with a centered fixation cross (1 s), followed by a picture presented for a maximum of 5 s or terminated by a response. It was then replaced by a blank screen (1 s) – as an ISI – before proceeding to the next trial. Prior to the recognition task, participants completed six practice trials (two I/R, two D, and two new).

The key-response mappings were counterbalanced across participants during recognition. For half of the participants, the keys “z” (labeled as “I/R”), “x” (labeled as “D”), and “/” (labeled as “N” for “new”) were assigned to be pressed with left middle and index fingers and the right index finger respectively; whereas for the other half of participants, the keys “/” (labeled as “I/R”), “.” (labeled as “D”), and “z” (labeled as “N” for “new”) were assigned to be pressed with right middle and index fingers and the left index finger, respectively. Participants were requested to rest their fingers on the corresponding keys and respond as quickly and accurately as possible.

Finally, the participants completed a battery of paper-and-pencil tests in the following order: the SCS, verbal skill test (i.e., the Shipley vocabulary test for Canadians and the vocabulary subtest of the Wechsler’s Adult Intelligence Scale for Chinese), the CES-D, the MMSE (for older adults only), and a demographic questionnaire. They were then debriefed and paid (or granted course credits). The demographic questionnaire and the instructions for the memory and VSWM tasks were translated and back-translated by two bilingual researchers who are fluent in both English and Mandarin. Any translation discrepancies were resolved through a follow-up discussion. All of the other paper-and-pencil tests have corresponding standardized Chinese versions.

## Results

To examine the effects of age and culture on memory performance, we conducted separate mixed-model ANOVAs on item memory and context memory scores. Timeout response rate was low (1% for the R/D block and 0.8% for the I/D block on average), and all the trials with a timeout response were excluded from the final analysis. Similar to a recent study on memory for items and their contexts [32], we adjusted for guessing in the calculation of both item and context memory scores by subtracting the false alarm from the corresponding hit rate.

### Item Memory

Item memory was measured with a corrected recognition score (i.e., item hit rate – item false alarm rate; see Table 2). For each item type (I/R or D), hit rate was the proportion of old items recognized as old, regardless of contexts; whereas false alarm rate was the proportion of new items recognized as old. Considering that meaningfulness ratings given to the R or I items and typicality ratings made to the D items differ in the nature and rating criterion, we ran separate analyses on each type of ratings. For meaningfulness ratings (i.e., R or I items), a 2 (age) × 2 (culture) × 2 (item type) mixed-model ANOVA revealed a significant main effect of age, *F*(1,139) = 23.29, *MSE* = 0.02, *p*<.001, η^2^ = 0.14, with better memory for young (*M* = 0.93, *SD* = 0.09) than for older adults (*M* = 0.85, *SD* = 0.09). None of the other effects reached significance (*p*s >.38). For typicality ratings (i.e., D items in the I/D or the R/D block), the age × culture × block ANOVA revealed a significant main effect of age, *F*(1,139) = 14.76, *MSE* = 0.03, *p*<.001, η^2^ = 0.10, with better memory for young (*M* = 0.91, *SD* = 0.09) than for older adults (*M* = 0.83, *SD* = 0.13). The main effect of item type was also significant, *F*(1,139) = 8.78, *MSE* = 0.01, *p*<.01, η^2^ = 0.06, with better memory for D items in the I/D block (*M* = 0.88, *SD* = 0.12) than for those in the R/D block (*M* = 0.86, *SD* = 0.14). None of the other effects reached significance (*p*s >.22). Separate analyses on hits and false alarms revealed a generally consistent pattern, with more hits and fewer false alarms made by young than by older adults, for D items in the I/D block than for those in the R/D block, respectively.

**Table 2:** Item Memory Performance.

		Canadian		Chinese	
		Young	Older	Young	Older
Corrected recognition	I	.93 (0.12)	.87 (0.11)	.93 (0.07)	.85 (0.09)
	R	.92 (0.12)	.86 (0.12)	.93 (0.06)	.84 (0.09)
	DI	.91 (0.12)	.84 (0.16)	.93 (0.06)	.85 (0.09)
	DR	.90 (0.12)	.80 (0.21)	.89 (0.09)	.84 (0.10)
Hits	I	.93 (.11)	.90 (.11)	.94 (.06)	.86 (.09)
	R	.92 (.12)	.88 (.11)	.94 (.06)	.86 (.09)
	DI	.92 (.12)	.89 (.12)	.94 (.06)	.87 (.09)
	DR	.91 (.12)	.86 (.18)	.92 (.09)	.87 (.10)
False Alarms	I	.01 (.02)	.03 (.04)	.01 (.02)	.02 (.03)
	DI	.00 (.01)	.03 (.04)	.01 (.02)	.02 (.05)
	R	.01 (.02)	.05 (.12)	.01 (.02)	.02 (.02)
	DR	.01 (.03)	.06 (.13)	.03 (.04)	.03 (.04)

*Note.* Each cell provides mean score, with standard deviation in parenthesis. I = Independent items; R = Relational items; DI = Daily Life items in the Independent block; DR = Daily Life items in the Relational block.

Taken together, young adults showed better item memory than older adults and this held true for both Chinese and Canadian samples. In addition, D items in the I/D block were better recognized than those in the R/D block.

### Context Memory

**Figure 2:**
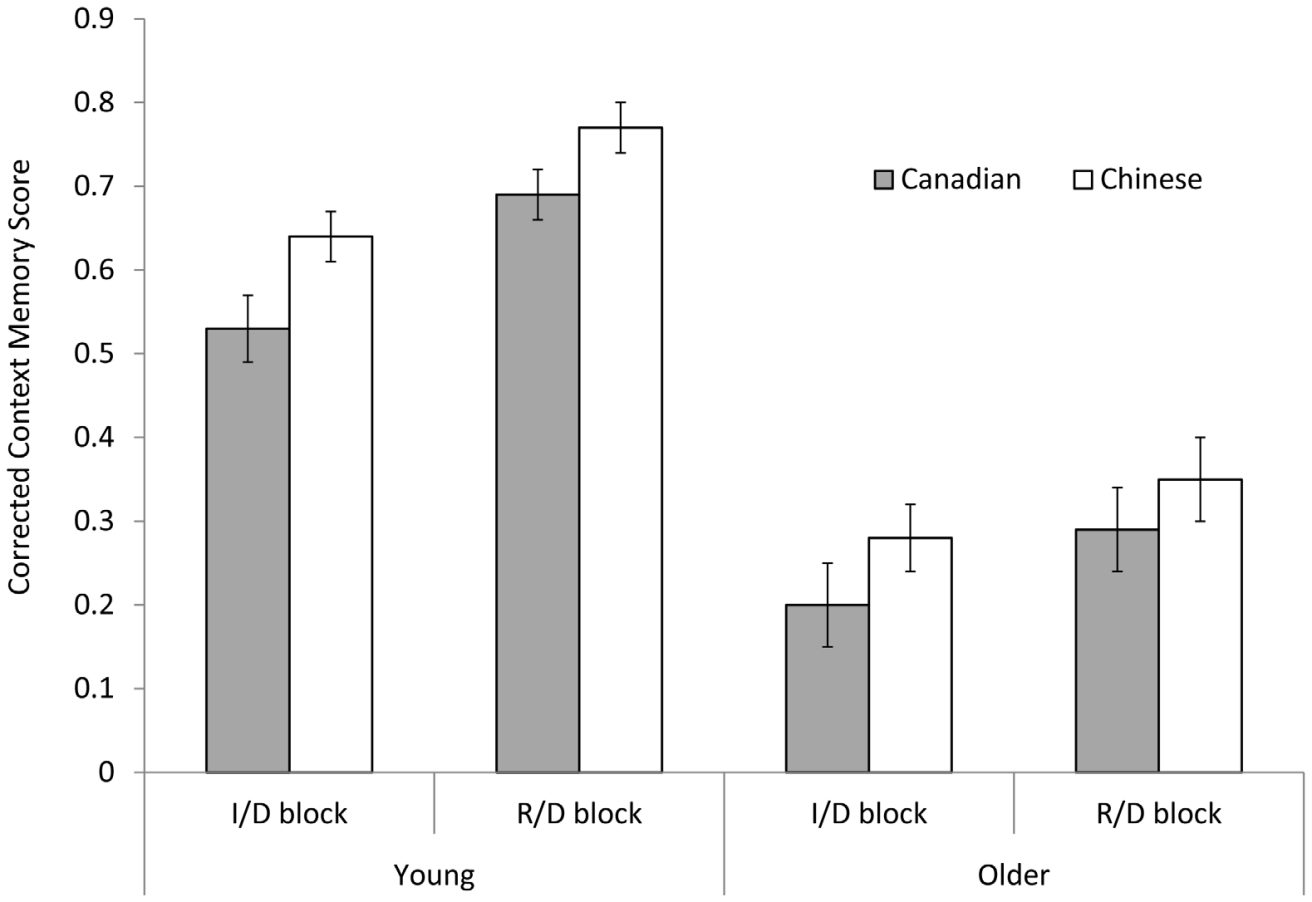
Corrected context memory scores across two memory blocks for the four age by culture groups. I/D = Independent vs. Daily Life Block; R/D = Relational vs. Daily Life Block.

Context memory refers to the ability to discriminate between the two contexts in this study. Considering the discrimination between the two contexts could be determined by both correct context attributions (i.e., context hits) and guesses or context misattributions (i.e., context false alarms), context memory was measured with a corrected score by adjusting for guessing. Separate context memory scores were calculated for the I/D and the R/D blocks (see Figure 2) by subtracting the context hit rate (i.e., the proportion of correct context attributions of targets) from the context false alarm rate (i.e., the proportion of context misattributions of lures). Taking the I/D block as an example (e.g., I items as targets and D items as lures), context memory score was calculated based on this formula: Context memory score = context hit rate – context false alarm rate = II/(II+DI) – ID/(DD+ID). In this formular, II = I items recognized as I (correct context attributions); DI = I items recognized as D; ID = D items recognized as I (context misattributions); and DD = D items recognized as D. The calculation taking D items as targets and I items as lures, DD/(DD+ID) – ID/(II+DI), produced the same results. The resulted context memory score reflects the ability to discriminate between I and D contexts.

The resulting context memory score indexes the ability to discriminate between items encoded in R or I context and those encoded in D context. The 2 (age)×2 (culture)×2 (block) mixed-model ANOVA on the corrected context memory score revealed significant main effects of culture, *F*(1,139) = 5.28, *MSE* = 0.10, *p*<.05, η^2^ = 0.04, age, *F*(1,139) = 104.82, *MSE* = 0.10, *p*<.001, η^2^ = 0.43, and block, *F*(1,139) = 25.31, *MSE* = 0.03, *p*<.001, η^2^ = 0.15. Context memory was better for Chinese (*M* = 0.51, *SD* = 0.28), young adults (*M* = 0.66, *SD* = 0.19), and the R/D block (*M* = 0.52, *SD* = 0.33) than for Canadians (*M* = 0.43, *SD* = 0.30), older adults (*M* = 0.28, *SD* = 0.25), and the I/D block (*M* = 0.42, *SD* = 0.31), respectively. None of the other effects were significant, *p*s >.15. Separate analyses on source hits and false alarms revealed a generally consistent pattern, with more hits and fewer false alarms by Chinese, by young adults, and in the R/D block than by Canadians, by older adults, and in the I/D block, respectively.

Taken together, the results of context memory analysis suggest a cultural effect favoring Chinese over Canadians, an age effect with young adults outperforming older adults, and an encoding block effect with an advantage for the R/D encoding relative to the I/D encoding.

## Discussion

The primary goal of this study was to examine cultural and age-related differences in memory for socially meaningful item-context associations. Most importantly, we found cultural differences in memory for socially meaningful item-context associations which favored Chinese over Canadians. This finding adds to reports of cultural differences in various cognitive domains, including perception, categorization, attention and memory [12], [33]–[35]. This finding is consistent with the literature suggesting that East Asians, driven by their holistic cognitive processing style, are more oriented towards processing and remembering meaningful contextual background [10], [11], [15]. However, this result is not consistent with two previous findings of no cultural differences in item-context binding [12], [13]. This may be because previous studies used incidental encoding of arbitrarily assigned item-source associations, whereas the current study engaged active and intentional encoding of socially meaningful item-context associations. In addition, the item-source binding in previous studies mainly involved perceptual features, whereas the current study engaged a semantically and socially meaningful binding. It is possible that memory for arbitrary, less meaningful, and incidentally encoded perceptual contexts primarily relies on biologically-based and thus culturally-invariant cognitive functions [14]. The current study adds to the existing literature by suggesting that binding items to social meaningful contexts in memory is influenced by cultural experience. The intentionally encoded socially meaningful item-context associations in the current study may promote context-dependent holistic processing style that is preferred by Chinese and thus differentially benefit their context memory.

This finding challenges the view that source memory is a basic, culture-invariant “hardware of the mind” [13], [36]. Of note, the cultural differences were specific to context memory but not found in item memory. We propose that socially meaningful item-context binding may recruit culturally-relevant knowledge, experiences, and/or strategies [14], and thus allows Chinese to engage in context-dependent encoding, which subsequently benefits their context memory. However, item memory was scored independently of item-context associations, and thus may primarily rely on basic biologically-driven cognitive functioning that is assumed to be the same across cultures [7], [14]. Along similar lines, it has been documented that older East Asians categorized less than their Western counterparts during recall of category exemplars, but the actual recall performance did not differ between the two cultures [35].

Interestingly, D items were better recognized in the I/D block than in the R/D block. A possible explanation for this finding is that the R context engaged more elaborative processing (relationship between the self and others) than did the I context (only the self), and consequently, encoding of D items was less effective in the context of R items than in the context of I items. This more complex and elaborative processing engaged for the R context may also explain the overall context memory advantage for the R/D block relative to the I/D block.

The substantial age effects on item and context memory performance replicated the well-established finding of age-related deficits in episodic memory (for reviews, see [37], [38]). In context memory, the results showed a cultural effect favoring Chinese and an age effect favoring young adults. The cultural effect and the age effect, however, were independent, suggesting that the context-dependent holistic processing strategies of Chinese did not ameliorate their age-related deficits in explicit memory binding. Although it has been suggested that adopting culturally-favored processing strategies may primarily rely on knowledge and/or experience-based pragmatics on which cultural effects tend to be magnified with aging [22], [35], the current study did not reveal this interaction. The absence of age by culture interaction has also been reported in free recall [35] and speaker source memory [13]. The current study extends this to memory for socially meaningful item-context associations. In the current study, the pragmatic-based processing strategies (for which cultural effects should be magnified with aging; Gutchess et al. [35] were applied to a basic resource-demanding item-context binding process (for which cultural effects should be reduced with aging [7]). This mutual counteraction may explain the absence of age by culture interaction.

It should be noted that the cultural effect on context memory appears to be context-independent rather than context-specific. Chinese participants outperformed Canadians in memory for not only their culturally favored relational (R) context but also for the independent (I) context that theoretically favors Canadians. This suggests that adopting culturally-favored context-dependent holistic processing of Chinese in the current context may primarily rely on knowledge and/or experience-based pragmatics that are independent of specific social contexts. Alternatively, it is also possible that although our samples were representative of their own cultural norms in terms of independent-interdependent self-construal, the I and R contexts may not have engaged the corresponding self-construal and thus did not show the context-specific cultural preference effects. In any event, further studies are needed to consolidate the conclusion.

Another limitation of the current work is that we did not include basic IQ measures to match the samples from the two cultures. The only cognitive measure included in the current study (i.e., VSWM) showed a cultural effect favoring Chinese over Canadians. This is presumably because the computerized VSWM task requires concrete visualization that differentially favors Chinese [39]. Upon a closer inspection, we noticed that two Canadian older adults scored 0 and three Chinese young adults scored 100 on the VSWM task. After excluding these five participants to artificially match the samples on VSWM (*p* = .11), the cultural effects on context memory remained marginally significant (*p* = .05). Furthermore, the samples from the two cultures did not differ in item memory and older adults from the two cultures did not differ on MMSE scores. Taken together, the reported cultural effect is not likely driven by sample differences in basic cognitive functions.

Despite these limitations, the current study introduced a novel socially meaningful item-context association manipulation that is sensitive to cultural effect. In doing so, the study provided the first empirical evidence for cultural effects on memory for socially meaningful item-context associations. Overall, Chinese remembered socially meaningful item-context associations better than Canadians. The cultural effect was well preserved into old age, suggesting that older Chinese continue to be able to benefit from their culturally-preferred processing style. This finding is insightful for potential advances of the currently dominant cultural convergence theory in the literature on culture and cognition [7].
